# Association between lipid accumulation product and non-alcoholic fatty liver disease with normal weight: a population-based study

**DOI:** 10.3389/fmed.2025.1565997

**Published:** 2025-08-20

**Authors:** Lingde Shen, Yuanfang Lin, Weifeng Chen, Hui Peng

**Affiliations:** ^1^Department of Tuina, Shenzhen Traditional Chinese Medicine Hospital (The Fourth Clinical College of Guangzhou University of Chinese Medicine), Shenzhen, China; ^2^Department of General Practice, Shenzhen Second People’s Hospital (The First Affiliated of Shenzhen University), Shenzhen, China

**Keywords:** lipid accumulation product, non-alcoholic fatty liver disease, normal weight, LAP, NAFLD

## Abstract

**Background:**

Previous studies have indicated that Lipid accumulation product (LAP) can serve as a predictor for various metabolic diseases. However, the relationship between LAP and Non-alcoholic fatty liver disease (NAFLD) in individuals with normal weight remains unclear.

**Methods:**

This study involves a secondary analysis of a retrospective cohort study conducted among a Japanese population consisting of 10,391 participants with normal weight, spanning from 2004 to 2015. LAP is determined by utilizing Waist circumference (WC) and Triglyceride (TG) levels. Participants were categorized into groups based on LAP quartiles. We performed an analysis of the association between LAP and NAFLD using Cox proportional-hazard regression, smooth curve fitting, and sensitivity analyses. Prediction accuracy was evaluated using the area under the curve (AUC) analysis, sensitivity, and specificity, using Receiver Operating Characteristic (ROC) curves.

**Results:**

The analysis revealed a statistically significant positive correlation between LAP and normal-weight NAFLD (*P* < 0.01). Following adjustment for various covariates in the full models, LAP was found to be associated with NAFLD (OR = 1.06, 95% CI: 1.04–1.08, *P* < 0.001). Sensitivity analysis was conducted by categorizing LAP as a quartile variable, showing that the top quartile had a 354% increased risk of NAFLD compared to the bottom quartile in the full model (OR = 4.54, 95% CI: 2.83–7.3, *P* < 0.001). A non-linear relationship was observed between the LAP and normal-weight NAFLD, with an inflection point identified at a LAP value of 12.6.Furthermore, ROC curve analysis demonstrated that LAP (AUC = 0.809, sensitivity = 83.3%, specificity = 64.7%) had superior predictive accuracy for normal-weight NAFLD compared to Body mass index (BMI) and WC.

**Conclusion:**

Our study reveals a non-linear association between the LAP and the risk of NAFLD in individuals of normal weight, with LAP demonstrating superior predictive accuracy compared to BMI and WC. These results advocate for the use of LAP thresholds to guide early lifestyle interventions during metabolically reversible stages and to improve the identification of high-risk lean individuals who may be overlooked by current BMI-based screening methods.

## Contributions to the literature

Despite the growing prevalence of Non-alcoholic fatty liver disease (NAFLD), the relationship between the Lipid accumulation product (LAP), a sensitive marker of metabolic dysfunction, and NAFLD in normal-weight individuals remains inconclusive. Conventional measures like BMI often fail to accurately capture obesity-related health risks. Emerging evidence, however, suggests that LAP is a robust predictor of metabolic syndrome in adults. This study aims to investigate the association between LAP and NAFLD in a cohort of normal-weight individuals, providing insights to inform the development of targeted public health policies and clinical interventions.

Non-alcoholic fatty liver disease is a prevalent chronic liver condition globally, ranging from simple steatosis to fibrosis (1–3). Its rising prevalence among young populations is particularly alarming due to associations with long-term adverse outcomes like cirrhosis, cardiometabolic syndrome, and increased risks of death or liver transplantation in adulthood (4, 5). Data from 1990 to 2006 shows a prevalence of NAFLD at 25.3%, which increased to 38.2% from 2016 to 2019, impacting approximately one-third of the global population (2). This surge presents a substantial challenge for health systems, underscoring the importance of screening the general population for NAFLD risk in order to tackle this escalating health issue (6).

The BMI is a crude marker of obesity that fails to account for the distribution of fat and lean tissue, leading to discrepancies in assessing obesity-related risks (7, 8). Hence, it is inapplicable for individuals categorized as metabolically obese but normal weight (MONW). It is observed that despite having a normal body mass index, these individuals exhibit a variety of obesity-related abnormalities, including dyslipidemia, insulin resistance, visceral obesity, type 2 diabetes and cardiovascular disease (9). In the general population, MONW individuals are at high risk for Metabolic syndrome (MS) (10). NAFLD, an obesity-related condition, is known as a hepatic manifestation of Metastatic Disease (11). Moreover, the presence of NAFLD in non-obese individuals increases the risk of metabolic abnormalities compared to obese individuals without NAFLD (12). Notably, the prevalence of NAFLD ranged from 15% to 21% in non-obese Asians with a BMI < 25 (13). According to Feng et al. (14), normal-weight individuals with NAFLD are more likely to develop diabetes, hypertension, and metabolic syndrome. This suggests that we need to pay closer attention to these individuals.

It has been found that LAP is one of the most significant predictors of MS in adults (15). Calculated based on TG levels and WC, LAP is a simple yet effective index for evaluating abdominal lipid accumulation (16). Compared to other indices, LAP has been found to perform better in monitoring and managing metabolic syndrome (17).

While previous studies have recognized the LAP as a marker for metabolic syndrome in general populations, our study is the first to provide large-scale evidence demonstrating LAP’s superior predictive value for NAFLD specifically in normal-weight individuals–a demographic often neglected in metabolic risk assessments. By concentrating exclusively on this understudied subgroup and controlling for obesity-related confounders, our findings reveal LAP’s unique capability to identify the risk of hepatic steatosis. This challenges conventional screening paradigms and offers a targeted approach for the early detection of NAFLD in lean populations.

## Materials and methods

### Data source and study design

The study design used in this research is a secondary analysis of an existing dataset. The current study is a cross-sectional analysis of data from subjects in the NAGALA study cohort. The study design and purpose of the NAGALA cohort have been previously described in detail (18, 19). In this research project, general population members receiving health services at Murakami Memorial Hospital have been recruited since 1994. In the research, examination data are analyzed to identify chronic diseases and their possible risk factors, as well as guide development of strategies to prevent chronic diseases. The Murakami Memorial Hospital Ethics Committee approved the NAGALA study, with informed consent obtained from participants (IRB2018-09-01). All participants provided written informed consent prior to enrollment, with particular emphasis on maintaining confidentiality throughout the processes of data collection, storage, and analysis. Personal identifiers were removed during dataset preparation, and anonymized health records were securely managed on password-protected institutional servers. Professor Okamura has uploaded the dataset to the Dryad database^1^ (18), thereby enabling additional researchers to utilize the study data for further analysis in compliance with the database’s terms. Between 2004 and 2015, a total of 20,944 individuals aged 18 and above were included in the initial research, having undergone a minimum of two regular medical check-ups at Murakami Memorial Hospital.

The original study’s exclusion criteria consisted of: (1) Patients with type 2 diabetes (*n* = 323),or individuals with fasting plasma glucose levels greater than 6.1 mmol/L (*n* = 808); (2) A total of 416 individuals who have been diagnosed with liver diseases, such as hepatitis C or B; (3) A total of 2321 individuals consumed medication; (4) A total of 739 individuals consumed excessive alcohol (more than 40 grams per day for women and more than 60 grams per day for men); (5) participants with incomplete data for covariates such as abdominal ultrasonography, laboratory measurements, or exercise and alcohol consumption (*n* = 863). Thus, the original dataset obtained contained 15,464 participants.

**FIGURE 1:**
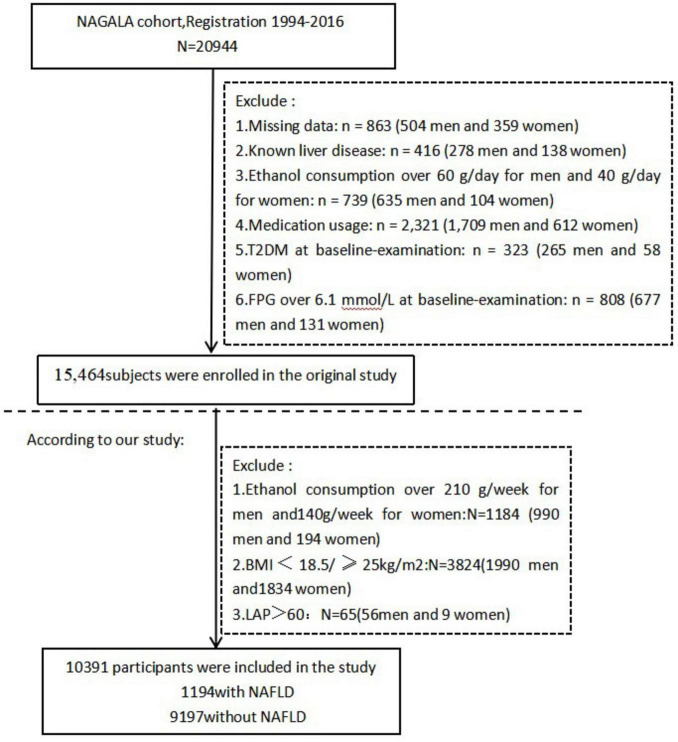
Flow diagram of subjects included in the cohort study.

For this secondary analysis, we further refined the original cohort by excluding 1,184 participants due to excessive alcohol consumption (defined as more than 210 g of alcohol per week for males and more than 140 g per week for females) (20). Additionally, participants with a BMI of less than 18.5 or equal to or greater than 25 kg/m^2^ (*N* = 3,824) and those with a LAP greater than 60 (*N* = 65) were excluded. Ultimately, a total of 10,391 participants were included in our study cohort (Figure 1).

### Definitions and calculations

Body mass index is calculated by dividing weight by height squared.

The LAP for men and women was calculated according to the respective formulas. For men, the formula is [WC (cm)−65] × TG concentration (mmol/L). For women, it is [WC (cm)−58] × TG concentration (mmol/L) (21). The waist circumference (WC) values below 65 cm for men were revised upward to 66.0 cm (*n* = 32) and those below 58 cm for women were revised upward to 59.0 cm (*n* = 23). This was to prevent non-positive LAP values, as suggested by Kahn (21). LAP was utilized as an exposure variable in the study.

### Ultrasonographic diagnosis of NAFLD

An abdominal ultrasound was utilized for the assessment of NAFLD, with gastroenterologists conducting a blinded review of the ultrasound images without access to participants’ personal information. Four ultrasound findings were analyzed to determine the final diagnosis: liver-to-kidney echo contrast, liver brightness, depth attenuation and vessel blurring (22). A new scoring system was applied to apparently healthy Japanese adults using ultrasonography. For diagnosing NAFLD, the AUC was 0.980, with a specificity of 100% and a sensitivity of 91.7% (95% confidence interval 87.0–95.1).

### Other variables

Self-administered questionnaires were used to obtain information about participants’ medical histories and lifestyle factors, such as alcohol consumption, physical activity and smoking habits (18).

The database file included the following variables (18):

Age, Sex, body weight, waist circumference (WC), gamma-glutamyltransferase (GGT), alanine aminotransferase (ALT), aspartate aminotransferase (AST), high-density lipoprotein cholesterol (HDL-C), total cholesterol (TC), triglyceride (TG), diastolic blood pressure (DBP), systolic blood pressure (SBP), fasting plasma glucose (FPG), hemoglobin A1C (HbA1c), exercise status, drinking status, and smoking status.

### Statistical analysis

Statistical significance was determined by a *P*-value of 0.05 using EmpowerStats software (X&Y solutions, Inc., Boston, MA, USA).^2^ The LAP quartiles were used to describe the baseline characteristics of the study population. Continuous variables with normal distributions are presented as mean + SD, while continuous variables with skewed distributions are displayed as median or interquartile range. A frequency and percentage chart was presented for categorical variables. Furthermore, the Kruskal-Wallis H test, one-way ANOVA and chi-square test were used to determine any differences among the groups. A multivariate logistic regression model with stepwise adjustments of non-collinear variables was used to determine the relationship between LAP and NAFLD. Odds ratios (ORs) with 95% confidence intervals (CIs) are presented in the results. Three models were constructed: model1 without adjustment, Model 2 adjusted for Sex and Age, and Model 3 adjusted for Sex, Age, BMI, AST, ALT, GGT, DBP, SBP, HbA1c, FPG, HDL-C, TC, exercise status, smoking status, and drinking status. Moreover, the ROC curve was used to determine the area under the curve, cut-off point, sensitivity, and specificity in assessing LAP for the recognition of NAFLD. Additionally, a study on threshold effects, in conjunction with smoothed curve fits, was carried out to investigate the correlation between LAP and NAFLD.

## Results

### Characteristics of the participants at baseline

Table 1 presents a summary of the characteristics of participants enrolled in the study categorized by quartiles of LAP. Participants with elevated LAP levels exhibited higher levels of BMI, Age, ALT, AST, GGT, FPG, DBP, SBP, HbA1c, and TC, while HDL-C levels were lower in groups with higher LAP values (all *P* < 0.05). The prevalence rates of NAFLD corresponding to LAP quintile grouping were as follows: Q1: 1.35%, Q2: 3.67%, Q3: 14.81%, Q4: 35.01%. A gradual rise in LAP levels was associated with an increase in NAFLD incidence.

**TABLE 1:** The Baseline Characteristics of participants.

LAP quartiles	Q1 (0.02–4.72)	Q2 (4.74–9.75)	Q3 (9.75–19.48)	Q4 (19.51–59.95)	*P*-value
Participants	2439	3246	2998	1708	
Age (years)	40.68 ± 8.47	42.97 ± 8.67	45.23 ± 8.70	47.29 ± 8.77	<0.001
BMI (kg/m^2^)	20.18 ± 1.23	21.28 ± 1.50	22.21 ± 1.51	23.13 ± 1.31	<0.001
ALT (IU/L)	15.15 ± 7.14	16.47 ± 8.26	19.50 ± 17.94	23.46 ± 13.01	<0.001
AST (IU/L)	16.70 ± 6.24	17.00 ± 6.14	18.07 ± 12.03	19.26 ± 7.12	<0.001
GGT (IU/L)	13.79 ± 8.42	15.97 ± 11.43	19.60 ± 16.28	25.76 ± 22.67	<0.001
HDL-C (mmol/l)	1.67 ± 0.38	1.56 ± 0.37	1.41 ± 0.35	1.18 ± 0.29	<0.001
TC (mmol/l)	4.77 ± 0.78	4.98 ± 0.81	5.25 ± 0.82	5.56 ± 0.84	<0.001
HBA1C (%)	5.11 ± 0.30	5.14 ± 0.30	5.19 ± 0.32	5.23 ± 0.34	<0.001
FPG (mmol/l)	4.98 ± 0.39	5.08 ± 0.39	5.20 ± 0.38	5.31 ± 0.38	<0.001
SBP (mmHg)	107.93 ± 12.47	111.18 ± 12.90	115.31 ± 13.61	118.76 ± 14.07	<0.001
DBP (mmHg)	66.84 ± 8.86	69.13 ± 9.30	72.00 ± 9.64	74.87 ± 9.73	<0.001
SEX					<0.001
Female	1502 (61.58%)	1754 (54.04%)	1279 (42.66%)	462 (27.05%)	
Male	937 (38.42%)	1492 (45.96%)	1719 (57.34%)	1246 (72.95%)	
Exercising status					<0.001
Not-regular exerciser	1925 (78.93%)	2641 (81.36%)	2490 (83.06%)	1429 (83.67%)	
Regular exerciser	514 (21.07%)	605 (18.64%)	508 (16.94%)	279 (16.33%)	
Smoking status					<0.001
Non-smokers	1741 (71.38%)	2133 (65.71%)	1696 (56.57%)	803 (47.01%)	
Ex-smoker	333 (13.65%)	538 (16.57%)	640 (21.35%)	405 (23.71%)	
Current-smoker	365 (14.97%)	575 (17.71%)	662 (22.08%)	500 (29.27%)	
NAFLD					<0.001
NO	2406 (98.65%)	3127 (96.33%)	2554 (85.19%)	1110 (64.99%)	
YES	33 (1.35%)	119 (3.67%)	444 (14.81%)	598 (35.01%)	
Alcohol consumption					<0.001
Non- consumer	2131 (87.37%)	2692 (82.93%)	2404 (80.19%)	1282 (75.06%)	
Light alcohol consumer	308 (12.63%)	554 (17.07%)	594 (19.81%)	426 (24.94%)	

Continuous data are expressed as mean ± SD. Categorical data are expressed as *n* (%). A *p*-value of < 0.05 was considered statistically significant. LAP, Lipid accumulation product; NAFLD, Non-alcoholic fatty liver disease; BMI, Body mass index; ALT, alanine aminotransferase; AST, aspartate aminotransferase; DBP, diastolic blood pressure; SBP, systolic blood pressure; GGT, gammaglutamyltransferase; HbA1c, hemoglobin A1c; HDL-C, high-density lipoprotein; TC, Total cholesterol; FPG, Fasting plasma glucose.

### The results of univariate analysis

The univariate analysis results, shown in Table 2, revealed that LAP, Age, BMI, ALT, AST, GGT, SBP, DBP, FPG, HbA1c, TC, smoking were positively associated with NAFLD. Conversely, HDL-C and exercising exhibited a negative correlation with NAFLD. Furthermore, the analysis indicated that men had a higher risk of developing NAFLD compared to women.

**TABLE 2:** The results of univariate analysis.

Variable	Statistics	OR (95% CI)	*P*-value
LAP	11.68 ± 9.68	1.10 (1.09, 1.11)	<0.001
Age (years)	43.79 ± 8.94	1.03 (1.02, 1.04)	<0.001
Sex			<0.001
Female	4997 (48.09%)	1.0	<0.001
Male	5394 (51.91%)	4.54 (3.91, 5.27)	<0.001
BMI (kg/m^2^)	21.59 ± 1.73	1.91 (1.83, 1.99)	<0.001
ALT (IU/L)	18.18 ± 12.73	1.08 (1.08, 1.09)	<0.001
AST (IU/L)	17.61 ± 8.47	1.05 (1.04, 1.06)	<0.001
GGT (IU/L)	18.12 ± 15.31	1.03 (1.02, 1.03)	<0.001
HDL-C (mmol/l)	1.48 ± 0.39	0.09 (0.08, 0.11)	<0.001
TC (mmol/l)	5.10 ± 0.85	1.53 (1.43, 1.63)	<0.001
HBA1C (%)	5.16 ± 0.32	3.75 (3.08, 4.55)	<0.001
FPG (mmol/l)	5.13 ± 0.40	5.47 (4.65, 6.43)	<0.001
SBP (mmHg)	112.85 ± 13.73	1.04 (1.03, 1.04)	<0.001
DBP (mmHg)	70.36 ± 9.76	1.06 (1.05, 1.06)	<0.001
**Exercising status**			
Not-regular exerciser	8485 (81.66%)	Ref	
Regular exerciser	1906 (18.34%)	0.82 (0.70, 0.97)	0.017
**Smoking status**			
Non-smokers	6373 (61.33%)	Ref	
Ex-smoker	1916 (18.44%)	2.08 (1.79, 2.41)	<0.001
Current-smoker	2102 (20.23%)	1.77 (1.52, 2.05)	<0.001
**Alcohol consumption**			
Non- consumer	8509 (81.89%)	Ref	
Light alcohol consumer	1882 (18.11%)	0.95 (0.81, 1.12)	0.562

Continuous data are expressed as mean ± SD. Categorical data are expressed as *n* (%). A *p*-value of < 0.05 was considered statistically significant. CI, Confdence interval; Ref, Reference; OR, Odds ratio; LAP, Lipid accumulation product; NAFLD, Non-alcoholic fatty liver disease; BMI, Body mass index; ALT, alanine aminotransferase; AST, aspartate aminotransferase; DBP, diastolic blood pressure; SBP, systolic blood pressure; GGT, gammaglutamyltransferase; HbA1c, hemoglobin A1c; HDL-C, high-density lipoprotein; TC, Total cholesterol; FPG, Fasting plasma glucose.

### The results of relationship between LAP and NAFLD

Table 3 presents the multiple adjusted associations between quartiles of LAP and NAFLD. We examined both non-adjusted and adjusted models. In Model 1, LAP showed a positive correlation with the incidence of NAFLD (OR = 1.10, 95% CI: 1.09–1.11, *P* < 0.001). The minimally adjusted model (adjusted for age and sex) yielded similar results (OR = 1.09, 95% CI: 1.08–1.10). The association remained significant after adjusting for the total model (Age, Sex, BMI, ALT, AST, GGT, DBP SBP, FPG, HbA1c, TC, HDL-C, exercise status, drinking status, and smoking status) (OR = 1.06, 95% CI: 1.04–1.08, *P* < 0.001). A sensitivity analysis was conducted using LAP as a categorical variable (quartiles). In the fully adjusted model, NAFLD risk increased 354% in the top quartile compared to the bottom quartile, with a significant trend across quartiles (*P* for trend < 0.001).

**TABLE 3:** Relationship between the LAP and NAFLD with normal weight in different models.

Variables	Model 1 OR (95% CI) *P*-value	Model 2 OR (95% CI) *P*-value	Model 3 OR (95% CI) *P*-value
LAP	1.10 (1.09, 1.11) < 0.001	1.09 (1.08, 1.10) < 0.001	1.06 (1.04, 1.08) < 0.001
**LAP quartile**			
Q1	Ref	Ref	Ref
Q2	2.77 (1.88, 4.09) < 0.001	2.54 (1.72, 3.76) < 0.001	1.33 (0.88, 2.01) 0.177
Q3	12.67 (8.86, 18.13) < 0.001	10.54 (7.35, 15.12) < 0.001	3.13 (2.08, 4.70) < 0.001
Q4	39.28 (27.47, 56.17) < 0.001	28.89 (20.10, 41.52) < 0.001	4.54 (2.83, 7.30) < 0.001
*P* for trend	<0.001	<0.001	<0.001

Multivariate Cox regression analysis proportional hazard models were employed to calculate Odds ratio (OR) and their 95% confidence intervals (CI) for assessing between the LAP and NAFLD. A *p*-value of < 0.05 was considered statistically significant. Model 1: we did not adjust for other covariants. Model 2: we adjusted for Age, Sex. Model 3: we adjusted for: Age, Sex, BMI, ALT, AST, GGT, SBP, DBP, FPG, HbA1c, TC, HDL-C, exercising status, smoking status and drinking status. CI, Confidence interval; Ref, Reference; OR, Odds ratio; LAP, Lipid accumulation product; NAFLD, Non-alcoholic fatty liver disease; BMI, Body mass index; ALT, alanine aminotransferase; AST, aspartate aminotransferase; DBP, diastolic blood pressure; SBP, systolic blood pressure; GGT, gammaglutamyltransferase; HbA1c, hemoglobin A1c; HDL-C, high-density lipoprotein; TC, Total cholesterol; FPG, Fasting plasma glucose.

### ROC analysis

**FIGURE 2:**
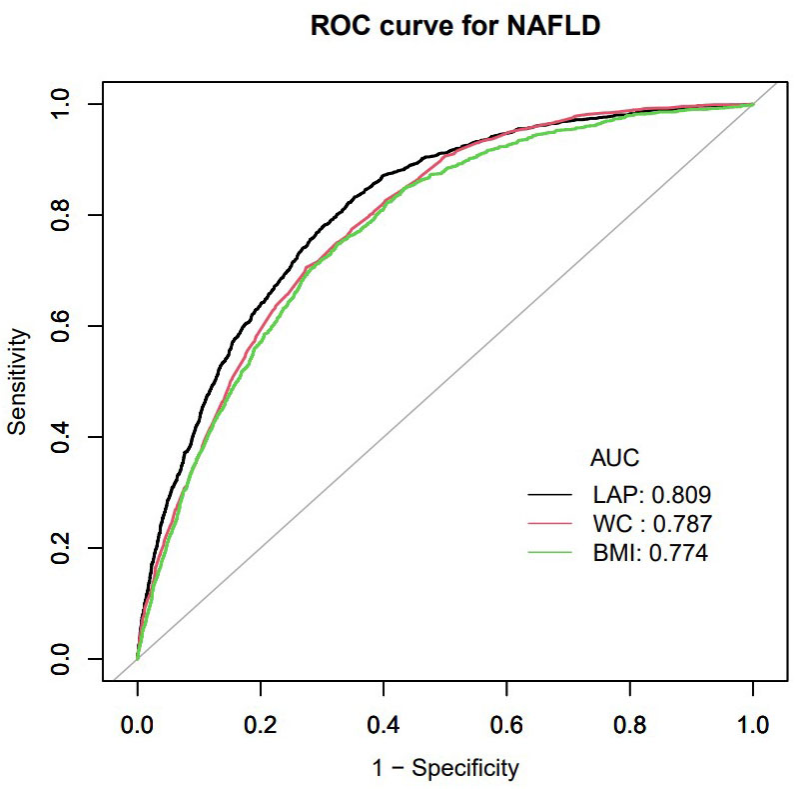
Receiver operating characteristic curve (ROC) in normal weight NAFLD population. AUC, area under the curve; LAP, Lipid accumulation product; BMI, Body mass index; WC, waist circumference; NAFLD, Non-alcoholic fatty liver disease.

Furthermore, researchers utilized an ROC curve to assess the predictive capabilities of LAP, BMI, and WC in determining the risk of NAFLD (Figure 2). The respective areas under the curve were as follows: BMI (0.774) < WC (0.787) < LAP (0.809) (Table 4). ROC curve analysis showed that LAP was a better predictor of normal-weight NAFLD than BMI and WC.

**TABLE 4:** Area under the curve (AUC) with the 95% CI of LAP, BMI, WC for predicting NAFLD

Variables	AUC	95% CI low blound	95% CI upp blound	Best threshold	Specificity	Sensitivity
LAP	0.809	0.798	0.821	10.926	0.647	0.833
WC	0.787	0.776	0.799	78.650	0.726	0.707
BMI	0.774	0.761	0.787	22.261	0.674	0.748

AUC, area under the curve; CI, confidence interval; LAP, Lipid accumulation product; BMI, Body mass index; WC, waist circumference; NAFLD, Non-alcoholic fatty liver disease.

### Results of a two-piecewise linear regression model

**FIGURE 3:**
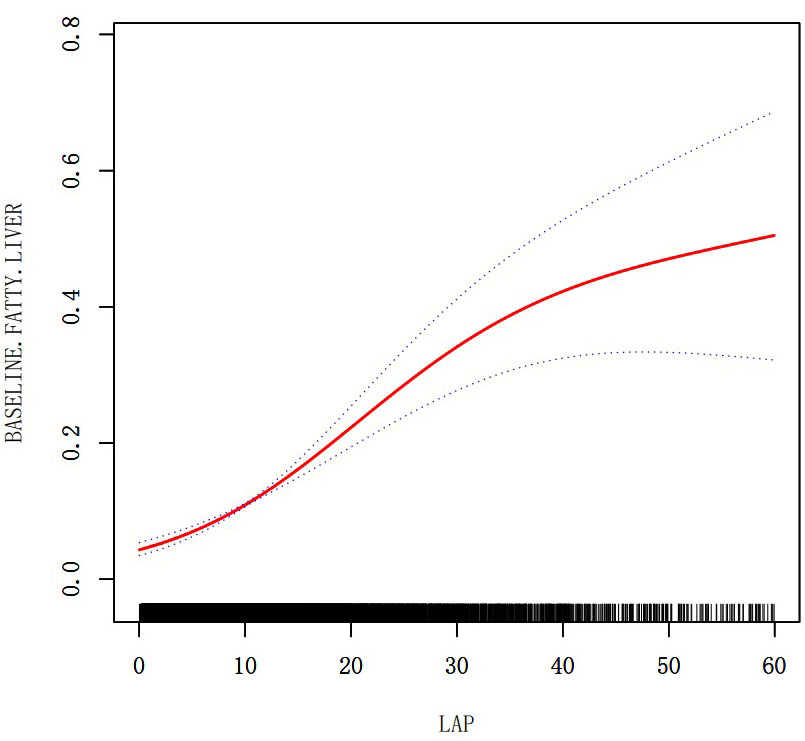
The relationship between LAP and NAFLD (adjusted for Age, Sex, BMI, ALT, AST, GGT, SBP, DBP, FPG, HbA1c, TC, HDL-C, exercising status, smoking status and drinking status).

A non-linear relationship between LAP and NAFLD was demonstrated through the use of cubic spline smoothing technique (Figure 3). In this study, the inflection point of LAP was determined to be 12.6 (Table 5), with a statistically significant disparity between the values on either side of this point (Log-likelihood ratio test *P* < 0.001). For LAP values ≤ 12.6, the risk of NAFLD increased by 1.19 times per unit increase in LAP (OR: 1.19, 95% CI: 1.15–1.24, *P* = 0.001). Conversely, for LAP values > 12.6, the risk of NAFLD increased by 1.05 times per unit increase in LAP (OR: 1.05, 95% CI: 1.03–1.06, *P* = 0.001).

**TABLE 5:** The result of two-piecewise linear regression model.

NAFLD	OR 95% CI	*P*-value
Fitting model by standard linear regression	1.06 (1.04, 1.08)	<0.001
**Fitting model by two-piecewise linear regression**		
Infection point of LAP	12.6	
≤12.6	1.19 (1.15, 1.24)	<0.001
>12.6	1.05 (1.03, 1.06)	<0.001
*P* for log likelihood ratio test	<0.001	

Two-segment Cox proportional hazards regression models were utilized to assess the data on each side of the inflection points. A *p*-value of < 0.05 was considered statistically significant. We adjusted for: Age, Sex, BMI, ALT, AST, GGT, SBP, DBP, FPG, HbA1c, TC, HDL-C, exercising status, smoking status and drinking status. CI, Confidence interval; OR, Odds ratio; LAP, Lipid accumulation product; NAFLD, Non-alcoholic fatty liver disease; BMI, Body mass index; ALT, alanine aminotransferase; AST, aspartate aminotransferase; DBP, diastolic blood pressure; SBP, systolic blood pressure; GGT, gammaglutamyltransferase; HbA1c, hemoglobin A1c; HDL-C, high-density lipoprotein; TC, Total cholesterol; FPG, Fasting plasma glucose.

## Discussion

This research found that elevated LAP was linked to a higher risk of NAFLD in Japanese individuals with normal weight (OR = 1.10, 95% CI: 1.09–1.11, *P* < 0.001), and there is a non-linear relationship between LAP and NAFLD in those of normal weight. When LAP was below 12.6, each standard deviation increase in LAP corresponded to a 19% higher NAFLD occurrence. When LAP exceeded 12.6, however, each standard deviation increase lead to only a 5% higher incidence of NAFLD, a smaller increase than for lower LAP values. ROC analysis revealed that LAP was a better predictor of NAFLD compared to BMI and WC. The results confirm that an elevated LAP significantly increases the risk of NAFLD.

Metabolically obese but normal weight individuals have a distinct metabolic profile compared to both metabolically healthy normal-weight and obese individuals with metabolic abnormalities (23), exhibiting a higher cardiovascular disease risk than healthy normal-weight individuals (24). Although lean NAFLD individuals may have a more favorable metabolic profile compared to obese NAFLD individuals, they still face a significantly higher risk for atherosclerotic cardiovascular disease (25). The presence of NAFLD in non-obese individuals increases the risk of metabolic abnormalities compared to obese individuals without NAFLD (12). Research has found LAP as a valuable index for recognizing metabolic alterations related to lipid accumulation status and predicting the onset of type 2 diabetes in non-obese individuals (26, 27).

This study revealed a relationship between the LAP and NAFLD with normal weight patients, which remained significant with and without adjusting for confounders. Additionally, the study found a non-linear relationship between LAP and NAFLD but identified an important inflection point at LAP 12.6. Prior to the inflection point (LAP ≤ 12.6), the metabolic system exhibits heightened sensitivity to lipid accumulation, characterized by adaptive mechanisms such as enhanced mitochondrial β-oxidation and suppressed *de novo* lipogenesis, which collectively maintain lipid homeostasis (28, 29). During this phase, interventions may exert a “leverage effect.” As illustrated in Table 1, the prevalence of NAFLD within the high LAP range is 35.01%, markedly higher than in the low LAP group. However, when LAP surpasses 12.6, although the absolute risk continues to escalate, the risk of NAFLD increases by only 5%, indicating a diminished sensitivity of the metabolic system to lipid accumulation and a deceleration in the rate of risk increase. This phenomenon may result from chronic lipid overload, leading to mitochondrial dysfunction, endoplasmic reticulum stress, and activation of inflammatory pathways, thereby inducing a state of lipotoxic adaptation in the liver and exacerbating liver disease (30–32). This two-phase risk pattern highlights the significance of LAP in identifying the “critical point” for implementing preventive measures to maximize effectiveness before irreversible metabolic harm takes place.

The pathogenesis of NAFLD involves an imbalance in lipid metabolism, leading to increased hepatic *de novo* lipid synthesis and reduced beta-oxidation of fatty acids, resulting in excessive fat accumulation in the liver (33–35). Dysregulated gene expression and abnormal lipid metabolism contribute to intracellular lipid accumulation and the progression of NAFLD (36). Additionally, the dysregulation of mitochondrial energetics in NAFLD disrupts the balance between lipid accumulation and disposal, leading to hepatic steatosis (37). LAP has been utilized as a tool to define NAFLD in individuals with limited alcohol intake (38). Excessive lipid accumulation in the liver is a hallmark of NAFLD, and LAP, along with indices like the hepatic steatosis index and fatty liver index, has been proposed to predict NAFLD in the general population (39). In conclusion, LAP plays a crucial role in defining and predicting NAFLD by reflecting the disrupted lipid metabolism balance that leads to excessive lipid accumulation in the liver, a hallmark of this condition.

The enhanced predictive accuracy of the LAP (AUC: 0.809) compared to BMI (AUC: 0.774) and WC (AUC: 0.788) for identifying NAFLD in individuals with normal weight is attributable to its comprehensive pathophysiological relevance. Unlike BMI, which is a rudimentary indicator of overall adiposity and does not account for fat distribution or metabolic dysregulation, LAP effectively integrates measures of central obesity (via waist circumference) and dyslipidemia (via triglycerides). This dual parameterization is essential for detecting MONW individuals, who maintain a normal BMI yet possess visceral adiposity and elevated triglyceride levels–critical factors contributing to hepatic lipid accumulation. While WC alone is indicative of abdominal adiposity, it does not capture the concurrent disturbances in lipid metabolism. In contrast, the LAP formula directly reflects the pathogenesis of NAFLD: visceral adipose tissue releases free fatty acids into the portal circulation, and hypertriglyceridemia indicates impaired lipid clearance and increased hepatic *de novo* lipogenesis. Consequently, in cases of NAFLD with normal BMI, LAP demonstrates superior predictive accuracy compared to BMI and WC.

The relationship between LAP and NAFLD exhibits significant variability across ethnicities and regions, influenced by genetic predispositions, metabolic phenotypes, and environmental factors. In northern China, the prevalence of NAFLD is elevated, likely due to a diet high in fat, whereas in southern China, the prevalence is comparatively lower, potentially due to the interaction between high-carbohydrate diets and the PNPLA3 gene (40). Additionally, research indicates that NAFLD prevalence is highest among Hispanic populations, who frequently possess the PNPLA3 rs738409 risk allele, with LAP demonstrating the greatest predictive efficacy in this group (41–43). Conversely, the prevalence of NAFLD is lowest among Black individuals, who tend to have subcutaneous fat distribution and carry the protective TM6SF2 variant, resulting in reduced diagnostic sensitivity of LAP (44). Although our Japanese cohort offers strong evidence for associations between LAP and NAFLD in normal-weight Asian populations, ethnic differences in NAFLD susceptibility require careful consideration when applying these findings across different populations.

This study constitutes a secondary analysis of original data, identifying LAP as a novel cross-sectional risk marker. Consequently, we are unable to ascertain whether elevated LAP levels precede the onset of NAFLD or are a result of hepatic steatosis, a distinction that is critical for comprehending disease mechanisms and developing prevention strategies. Furthermore, our study does not elucidate the relationship between fluctuations in LAP and the progression or regression of fibrosis, which is essential for effective disease monitoring and the customization of treatment approaches. Additionally, we are unable to evaluate the long-term risk associated with LAP or its predictive value for outcomes such as cirrhosis or mortality. Future research should aim to elucidate the longitudinal role of LAP, with a particular focus on monitoring changes in LAP both before and after the onset of NAFLD. Also, it is important to quantify LAP’s predictive value for fibrosis progression and to evaluate interventions guided by LAP levels. However, our findings offer significant insights into the non-linear relationship between the LAP and normal-weight NAFLD. LAP demonstrates superior predictive accuracy for normal-weight NAFLD compared to BMI and WC. It facilitates early identification during the critical pre-disease phase (LAP ≤ 12.6), where lifestyle interventions may prevent the progression to NAFLD. Moreover, public health initiatives could integrate LAP into electronic health record alerts and occupational health screenings to identify at-risk normal-weight individuals.

Some of the advantages of our study are as follows: (1) For the first time, the article identified a correlation between LAP and NAFLD in normal-weight individuals, providing new insights into NAFLD risk management. (2) Due to its ease of calculation, LAP makes sense for practical applications. Furthermore, the NAGALA project’s samples represent a large general health check-up population, which makes our findings relevant for public health promotion. We present herein a new tool for monitoring and preventing in patients with NAFLD who are normal weight.

There are a number of limitations to this study that should be acknowledged. Despite excluding all reported medication users, our study was unable to account for the use of over-the-counter drugs or medication adherence. Additionally, while adjustments for lifestyle factors partially mitigate confounding related to socioeconomic status, future prospective studies should incorporate direct measures of education, income, and healthcare access to comprehensively characterize these relationships. Our exclusion criteria, while facilitating a focused investigation of NAFLD in normal-weight individuals, restrict the generalizability of the findings to other populations. The BMI range of 18.5–25 kg/m^2^ excludes overweight and obese individuals, and the LAP ≤ 60 criterion excludes those with severe lipid accumulation. This approach allows for a precise characterization of LAP’s role in the early development of NAFLD. Future studies should aim to validate these findings across a broader range of BMI and metabolic conditions. Another limitation of the article is the use of abdominal ultrasonography for diagnosing NAFLD, which may underestimate liver fat content in certain individuals (45). Furthermore, the term Metabolic dysfunction-associated steatotic liver disease (MASLD) is increasingly utilized to highlight the metabolic factors contributing to hepatic steatosis. Our study’s retrospective data set lacks key cardiovascular metabolic risk factors for diagnosing MASLD, like insulin resistance and C-reactive protein, and excludes diabetes patients and drug users. We used the traditional NAFLD definition via ultrasound. Future studies should adopt the MASLD criteria for better alignment with current metabolic liver disease classifications and risk stratification in normal-weight individuals.

## Conclusion

A non-linear association is observed between the Lipid Accumulation Product LAP and normal-weight NAFLD, characterized by a significant positive correlation when LAP values are below 12.6. Furthermore, LAP exhibits greater predictive accuracy for normal-weight NAFLD in comparison to BMI and WC. These findings support the implementation of LAP thresholds as a tool to guide early lifestyle interventions and enhance the identification of high-risk lean individuals who may not be detected through BMI-based screening methods.

## Data Availability

Publicly available datasets were analyzed in this study. This data can be found here: Data can be downloaded from the “DATADRYAD” database (https://datadryad.org/) (https://doi.org/10.5061/dryad.8q0p192).

## Footnotes

1
https://doi.org/10.5061/dryad.8q0p192

2
http://www.empowerstats.com/cn/
